# Application of a Sensitive Capture Sequencing Approach to Reservoir Surveillance Detects Novel Viruses in Zambian Wild Rodents

**DOI:** 10.3390/v16111754

**Published:** 2024-11-09

**Authors:** Lavel C. Moonga, Jones Chipinga, John P. Collins, Vishal Kapoor, Ngonda Saasa, King S. Nalubamba, Bernard M. Hang’ombe, Boniface Namangala, Tapiwa Lundu, Xiang-Jun Lu, Samuel Yingst, J. Kenneth Wickiser, Thomas Briese

**Affiliations:** 1 Department of Paraclinical Studies, School of Veterinary Medicine, University of Zambia, Lusaka 10101, Zambia; lavel.moonga@unza.zm (L.C.M.); mudenda68@yahoo.com (B.M.H.); b.namangala@unza.zm (B.N.); 2 Africa Centre of Excellence in Infectious Diseases of Humans and Animals (ACEIDHA), School of Veterinary Medicine, University of Zambia, Lusaka 10101, Zambia; 3 Vaughan Regional Medical Center, Selma, AL 36701, USA; chipingajoe2019@gmail.com; 4 Global Alliance for Preventing Pandemics at the Center for Infection and Immunity, Mailman School of Public Health, Columbia University, New York, NY 10032, USA; jc5966@cumc.columbia.edu (J.P.C.); vk2040@cumc.columbia.edu (V.K.); xl2134@columbia.edu (X.-J.L.); sy3229@cumc.columbia.edu (S.Y.); jkw2161@cumc.columbia.edu (J.K.W.); 5 Department of Zoology, Rabindranath Tagore University, Bhopal 464993, India; 6 Department of Disease Control, School of Veterinary Medicine, University of Zambia, Lusaka 10101, Zambia; nsaasa@gmail.com; 7 Department of Clinical Studies, School of Veterinary Medicine, University of Zambia, Lusaka 10101, Zambia; king.nalubamba@unza.zm; 8 Department of Biomedical Sciences, School of Veterinary Medicine, University of Zambia, Lusaka 10101, Zambia; tapiwalundu@gmail.com; 9 Department of Population and Family Health, Mailman School of Public Health, Columbia University, New York, NY 10032, USA; 10 Department of Epidemiology, Mailman School of Public Health, Columbia University, New York, NY 10032, USA

**Keywords:** pre-pandemic surveillance, One Health, pathogen discovery, adenovirus, chaphamaparvovirus, paramyxovirus, jeilongvirus, kobuvirus, aichivirus, next generation sequencing

## Abstract

We utilized a pan-viral capture sequencing assay, VirCapSeq-VERT, to assess viral diversity in rodents from the Eastern Province of Zambia as a model for pre-pandemic viral reservoir surveillance. We report rodent adeno-, parvo-, paramyxo-, and picornaviruses that represent novel species or isolates, including murine adenovirus 4, two additional species in the genus *Chaphamaparvovirus*, two paramyxoviruses distantly related to unclassified viruses in the genus *Jeilongvirus*, and the first *Aichivirus A* sequence identified from rodents in Africa. Our results emphasize the importance of rodents as a reservoir for potential zoonotic viruses.

## 1. Introduction

Rodents and shrews constitute two large orders of mammals that contain diverse species with widespread geographic distribution and ecology. These small animals have been established as reservoirs for a range of viruses and are linked to many human diseases. Hantaviruses and arenaviruses are two groups of zoonotic viruses of concern that are harbored by rodents and cause severe viral hemorrhagic fever. Both hantaviruses and arenaviruses are known to cause chronic infections in rodents, which shed the virus in urine, feces, and saliva. Humans can then become infected by contact with contaminated surfaces and desiccated excreta. Since the discovery of Lassa virus in 1969, no novel hemorrhagic fever-associated arenaviruses have been reported from Africa until Lujo virus (LUJV) was identified in 2008 [1,2]. It was discovered in South Africa during a nosocomial outbreak and transmission of human disease with high case fatality [3]. Despite multiple efforts, the reservoir of the virus has not been determined. Previous attempts to identify a reservoir for Lujo virus had shown the circulation of several arena- and paramyxoviruses in Zambian murine and shrew species [4,5,6]. The 2014 Zambian study analyzed over 400 wild rodents and 31 wild shrews collected across four locations in Zambia and reported various paramyxoviruses from different species of rodents. Paramyxoviruses are large, enveloped RNA viruses with a negative-sense, non-segmented genome [7]. The identified viruses were related to members of the paramyxovirus genera *Morbillivirus*, *Narmovirus*, and *Henipavirus* and the Tailam, Beilong, and J viruses of the genus *Jeilongvirus*. Jeilongviruses have been observed in rodents in Asia and Australia but were later also reported in rodents in Africa [8,9,10].

Similarly, knowledge about the prevalence, ecology, and phylogeny of adenoviruses, parvoviruses, or picornaviruses in rodents and shrews from Africa is limited. Adenoviruses are non-enveloped, icosahedral DNA viruses with a linear, double-strand genome [11]. They infect humans and animal species with infections ranging from asymptomatic to mild to severe fatal diseases. Adenoviruses are mostly considered to be species-specific, but cross-species transmission is possible, and serological findings suggest wider host ranges for some adenoviruses, e.g., canine adenovirus may also infect wolves, walruses, black and polar bears, and fishers [12,13,14]. There are currently three recognized murine adenovirus species: *Murine mastadenovirus A* (represented by murine adenovirus 1; MAdV-1), *Murine mastadenovirus B* (murine adenovirus 2; MAdV-2), and *Murine mastadenovirus C* (murine adenovirus 3; MAdV-3). Between them, MAdV-2 is divergent from the more closely related MAdV-1 and -3 [15,16]. Circulation of adenoviruses in multiple African wild rodent species has been reported in three studies that indicated a relationship to MAdV-2 based on short, PCR-generated sequences [17,18,19].

Parvoviruses are small, non-enveloped DNA viruses with a linear single-strand genome that cause disease in humans and other animal species [20]. Pathogenic mouse kidney parvovirus (MKPV) has been described in laboratory mouse strains [21,22,23,24], and murine chapparvovirus (MuCPV) in free-ranging urban house mice (*Mus musculus*) with undetermined pathology [25]; both are viruses of the species *Chaphamaparvovirus rodent1* in the genus *Chaphamaparvovirus*. From wild rodents there is only an additional ~1kb NS1 sequence from a house mouse in China reported [26], and we are not aware of reports from wild rodents in Africa.

Aichiviruses are small, non-enveloped, icosahedral RNA viruses with a positive-sense, non-segmented genome and are classified into several species in the genus *Kobuvirus* [27]. Human Aichi virus (aichivirus A1, species *Aichivirus A*) is separated into three genotypes, A–C, that can cause gastroenteritis and other forms of enteric disease, particularly in children or immuno-compromised individuals [28,29] The virus is transmitted by the fecal–oral route and frequently reported from wastewater or contaminated foods, especially shellfish. The species comprises additional viruses from canines, felines, birds, and rodents (currently nine ‘types’, aichivirus A2 to A10), including murine kobuviruses (A3/A8), rat kobuviruses (A6/A9), vole kobuvirus (A7), and bat kobuvirus (A10). Although there are some reports on kobuviruses in rodents from Asia, the USA, and Europe, we are not aware of any on rodents in Africa [25,30,31,32,33,34,35,36,37,38].

We analyzed tissues from rodents trapped in the Katete district of eastern Zambia using a novel capture sequencing assay that has the potential for more efficient virus detection and discovery compared to prior approaches. VirCapSeq-VERT is a sample and pathogen-agnostic approach shown to detect all vertebrate viruses as well as novel viruses not known at the time of assay design [39]. VirCapSeq-VERT uses a set of 1 million biotinylated oligonucleotides to enrich for viral sequences [40]. These biotinylated capture oligonucleotides are hybridized to conventionally prepared sequencing libraries, then trapped by magnetic streptavidin beads, washed, and the thus virus-enriched material is finally subjected to high-throughput sequencing.

## 2. Material and Methods

### 2.1. Rodent Trapping and Sample Collection

Rodents (*n* = 118) were trapped at four sites, namely: Azeleguza, Boma, Kachipu, and Nyembe in the Katete district in the Eastern Province of Zambia (14°5′42″ S, 32°2′13″ E) with permission from the local leadership and owners of the sampling fields. The animals were captured around fields using Sherman traps. Liver, kidney, spleen, and/or lung tissue were collected from anesthetized and then euthanized animals. The tissue samples were homogenized in Minimum Essential Medium and stored at −80 °C before they were transferred to the Center for Infection and Immunity (CII) for sequencing with approval from the Zambian National Health Research Ethics Committee (E0013022) and the Ministry of Fisheries and Livestock.

Rodent species were identified through the nucleotide sequence of the mitochondrial cytochrome b gene [4]. Sequences and species were identified through BLAST analysis and alignment to GenBank reference sequences. For four animals (3%), the species could not be identified due to a lack of suitable sequence information.

### 2.2. VirCapSeq-VERT High-Throughput Sequencing

Details on the VirCapSeq-VERT method, including probe selection strategy, listing of taxa targeted, and performance validation, have been published previously [39,40]. Briefly, total nucleic acids were extracted on the EasyMag platform (Biomerieux). All collected tissues were extracted, and available nucleic acid extracts from tissues of the same animal were subsequently pooled for sequence analysis. First- and second-strand cDNA was synthesized using Superscript IV (Invitrogen, Waltham, MA, USA) with random hexamer priming (Thermo Fisher Scientific, Waltham, MA, USA) and Klenow enzyme (New England Biolabs, Ipswich, MA, USA). Libraries were prepared with the Twist Library Preparation EF 2.0 reagent kit and barcoded with Twist Unique Dual Index primers, pooled, and hybridized in Twist Fast Hybridization buffer to the custom VirCapSeq-VERT probe set (Twist Biosciences, South San Francisco, CA, USA). After washing and subsequent amplification, the captured libraries enriched for viral sequences were analyzed on the Illumina NextSeq2000 system using an Illumina High Output P2 200-cycle cartridge and a read length of 150 bases.

### 2.3. Bioinformatic Analyses

Bioinformatic analyses of sequence data were performed using the Rapid Identification of Microbes (RIM) bioinformatics pipeline. FASTQ data were trimmed, QC filtered, and run statistics displayed (fastp, https://github.com/OpenGene/fastp, accessed on 3 September 2024; falco, https://falco.readthedocs.io/en/latest/, accessed on 3 September 2024; MultiQC, https://multiqc.info/, accessed on 3 September 2024). After removal of host sequences (Kraken2 suite, https://ccb.jhu.edu/software/kraken2/, accessed on 3 September 2024), reads were de novo assembled (SPAdes, https://github.com/ablab/spades, accessed on 3 September 2024) and, together with remaining singletons, searched for homology to database references (MegaBLAST; custom version of RVDB, https://rvdb.dbi.udel.edu/, accessed on 3 September 2024). Results were transformed into Excel tables that can be sorted and filtered. In the interactive screen interface of RIM, the results are also displayed ranked by bit-score with links to a reference sequence and pairwise alignment, and the number of reads and percentage of reference sequence coverage are listed. Mapping results in standard IGV format with links to the generated .bam and .bai files and the consensus sequence (SAMtools, http://www.htslib.org/, accessed on 3 September 2024) are also provided for download.

The IQtree package was used for inferring phylogenetic relationships [41]. Pairwise distance matrix calculations were performed using the Needleman–Wunsch global alignment algorithm with standard settings [42].

## 3. Results

In a survey of rodents for vertebrate virus infection in Zambia, we studied 118 animals from four locales in the Katete district. The samples included animals from the rodent species *Mastomys natalensis* (*n* = 105), *Aethomys chrysophilus* (*n* = 5), and *Rattus rattus* (*n* = 3), and one animal from the shrew species *Crocidura hirta*. Nucleic acid extracts of tissues (kidney, lung, liver, and/or spleen) from an individual animal were pooled and analyzed by VirCapSeq-VERT, generating an average of 11 million reads per sample (Supplementary Table S1). Viral sequences were identified in 98% of animals (116/118) and represented in addition to retroviruses viruses from six families (*Adenoviridae*, *Paramyxoviridae*, *Papillomaviridae*, *Parvoviridae*, *Picornaviridae*, and *Polyomaviridae*), including sequences virtually identical to known rodent viruses such as Mastomys coucha papillomavirus, Mastomys natalensis polyomavirus, or murine adeno-associated virus (Table 1, Supplementary Table S2). The data also included divergent sequences indicative of novel adeno-, paramyxo-, and parvoviruses and the first identification of murine kobuvirus from wild rodents in Africa.

### 3.1. Murine Adenovirus from Zambia

**Figure 1:**
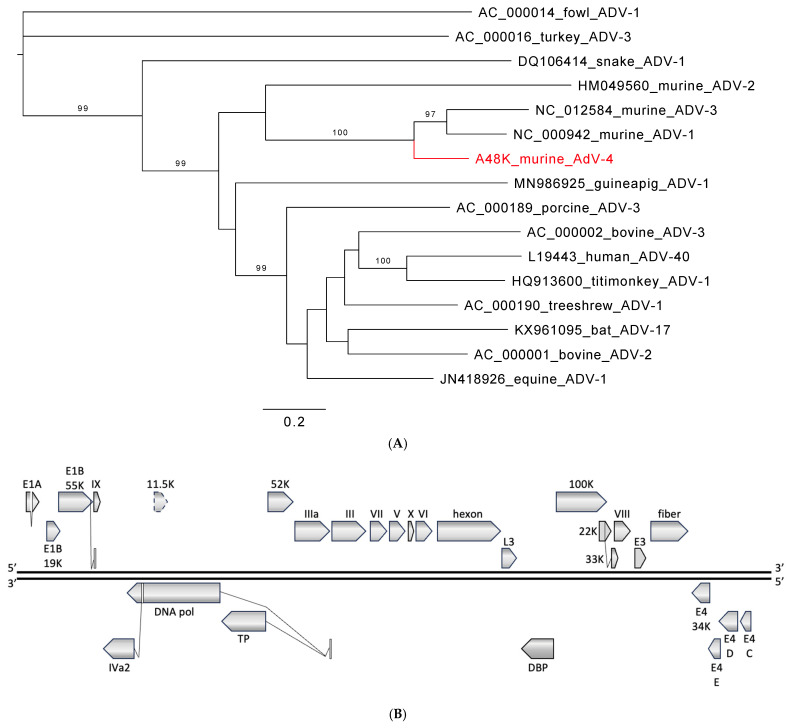
Murine adenovirus from Zambia. (**A**) Phylogenetic relationship to selected viruses in the genus *Mastadenovirus* based on the polymerase amino acid sequence. Phylogeny was reconstructed with the maximum likelihood method applying a Q.pfam+F+I+G4 substitution model as selected by Model Finder implemented in IQtree; bootstrap values (>85%) resulting from 1000 pseudoreplicates are indicated at the respective nodes; the scale bar indicates the number of amino acid substitutions per site, and GenBank accession numbers are given next to the branches. The red font indicates a virus described in this study. (**B**) Schematic of genome organization. Predicted open reading frames (ORF) and potential splice sites analogous to the other murine adenoviruses are indicated for both strands in all three frames. An ORF with 67% amino acid identity to an 11.5 kDa ORF in the same genome position of HAdV-41 (and other mastadenoviruses) that is not found in the other MAdV is also indicated.

Analysis of sequence data indicated two virtually identical 31 kb contiguous sequences (contigs) from the samples of two multimammate mice (*Mastomys natalensis*). BLASTn analysis showed very limited homology of <78% to adenoviral GenBank entries over short stretches of <1 kb. Analyses of translated amino acid sequences revealed a relationship between the novel sequence and members of the species *Murine mastadenovirus C* and *Murine mastadenovirus A* (genus *Mastadenovirus*, family *Adenoviridae*) (Figure 1A). As is common in mastadenoviruses, open reading frames (ORFs) *IVa2*, *DNA pol*, *TP*, and *33K* contain deduced splice sites (Figure 1B). Splice sites matching those of the related MAdVs are also present in *E1A* and *E1B*. However, the site analogous to the terminal donor site in *E1A* of MAdV-1 is positioned after the preceding stop codon of the *E1A* ORF, like in MAdV-3. There is no premature stop of E1B 19K as in MAdV-3, but a contiguous ORF as in MAdV-1. In contrast to the two start codons for 22K/33K in MAdV-3, only one methionine is present as in MAdV-1. In the most variable adenoviral region, E3, a single ORF exists as in MAdV-1 and -3, but with only limited local homology (43–55% identity). ORFs C, D, and E of the E4 region are present (45–80% identity with MAdV-1 and -3), but ORF A (present in MAdV-1 and -3) and ORF B (present in MAdV-1 but not -3) are both missing. Also, the nucleotide composition differs and is higher (59.3% GC) than for MAdV-1 (47.8% GC) and MAdV-3 (47.2% GC), and lower than for MAdV-2 (63.4% GC). Finally, the polymerase amino acid sequence distance was >15% from the three known murine adenoviruses (Supplementary Table S3). Based on these features, differing nucleotide composition, genome organization, and polymerase amino acid sequence, the Zambian murine adenovirus represents a fourth murine adenovirus, MAdV-4.

A partial sequence of MAdV-4 was detected in another eight multimammate mice (8%, 10/118; 3–59% genome coverage) (Table 1).

### 3.2. Rodent Chaphamaparvoviruses from Zambia

**Figure 2:**
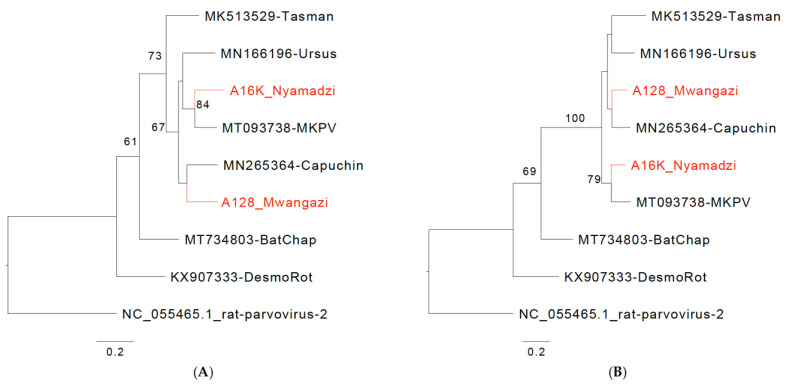

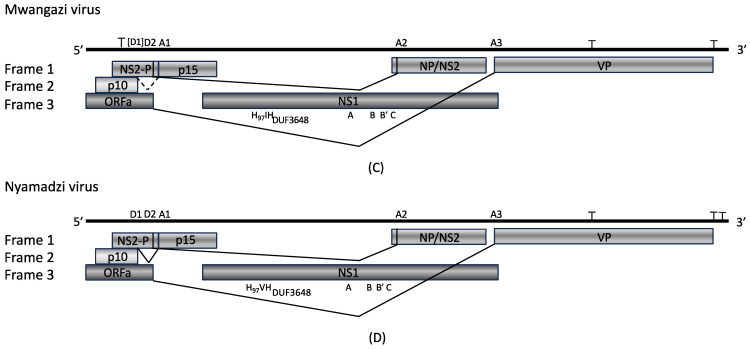
Chaphamaparvoviruses from Zambia. Phylogenetic relationship of Mwangazi and Nyamadzi viruses to other viruses in the genus *Chaphamaparvovirus* based on NS1 (**A**) and VP (**B**) amino acid sequences. Phylogeny was reconstructed with the maximum likelihood method by applying a JTT+G4 substitution model for NS1 and a Q.yeast+F+G4 substitution model for VP1, selected by Model Finder implemented in IQtree; bootstrap values (>60%) resulting from 1000 pseudoreplicates are indicated at the respective nodes; the scale bars indicate the number of amino acid substitutions per site, and GenBank accession numbers are given next to the branches. The red font indicates viruses described in this study. (**C**) Schematic of Mwangazi virus genome organization. (**D**) Schematic of Nyamadzi virus genome organization. Gray shading indicates the three possible reading frames. Predicted major splice sites (donor sites D1, D2, and acceptor sites A1–A3), polyadenylation signals (T), SF3 helicase (H97), Walker A, B, B’, C, and domain of unknown function (DUF) 3648 motifs are indicated.

We identified two 4 kb contigs with BLASTn homology of approximately 80% to murine chaphamaparvoviruses that branched in phylogenetic analyses separate from classified species (Figure 2A,B). Genomic features were analogous to those of mouse kidney parvovirus (MKPV; *Chaphamaparvovirus rodent1*), capuchin kidney parvovirus (CKPV, *Chaphamaparvovirus primate1*), and Tasmanian devil-associated chapparvovirus 2 (TdChPV2, *Chaphamaparvovirus dasyurid2*), which are species in the genus *Chaphamaparvovirus* (subfamily *Hamaparvovirinae*, family *Parvoviridae*). A common feature of these viruses is a 5′ p10 ORF that is also present in the viruses from Zambia. Based on NS1 and VP1 analyses, these viruses are also close to *Ursus americanus* parvovirus (UaPV; *Chaphamaparvovirus carnivoran3*) and *Ursus thibetanus ussuricus* chapparvovirus (UtPV; not classified) (Figure 2A,B), but detailed analysis is hindered by the 5′ truncated sequence for these viruses (GenBank Accession NC_077031 and OR779981). Both Zambian viruses, named Mwangazi virus and Nyamadzi virus, include the SF3 helicase family signature motifs Walker A, B, B’, and C in NS1 [43,44] and a domain of unknown function (DUF) 3648 described for NS1 of Brazilian bat chaphamaparvoviruses (Figure 2C,D) [45]. Like other chaphamaviruses, they lack a PLA2 domain in VP that is found in other parvovirus genera [46]. Two major splice donor sites (D1, D2) and three acceptor sites (A1–A3) have been experimentally mapped for MKPV [22]. In Mwangazi and Nyamadzi viruses, A1–A3 and D2 appear largely conserved. The region of D1 shows indel and sequence variation between the viruses, and two deduced D1 sites appear possible in the Nyamadzi virus, the second one with better conservation (Supplementary Table S4). In the Mwangazi virus, the analogous site does not conform with the canonical consensus, and another canonical site is located 5′ of the p10 termination codon so that a p10/p15 fusion protein would be generated that is not observed in the other viruses (D1a, Supplementary Table S4). The region between D1 and A1 differs in additional aspects between the viruses. NS2-P and p15 in Mwangazi and Nyamadzi viruses constitute one continuous ORF, and expression of p15 functionality may not require efficient D1/A1 splicing, whereas splicing may be essential in the other viruses where NS2-P and p15 are in different frames (MKPV, CKPV) or separated by a stop codon (MuCPV, TdChPV2). Based on the NS1 amino acid identity of 74% between each other and less than 85% with the existing species, both viruses qualify as novel species in the genus *Chaphamaparvovirus* (Supplementary Table S5) [47].

Mwangazi virus was more prevalent and found in 22 multimammate mice (19%, 22/118; 9–100% genome coverage). Nyamadzi virus was present in 4 animals (3%, 4/118; 80–100% genome coverage): 3 black rats (Rattus rattus) and one multimammate mouse (Table 1). For both viruses, minor sequence variation between genomes was observed, comparable to what has been reported for MKPV [22].

### 3.3. Rodent Jeilongviruses from Zambia

**Figure 3:**
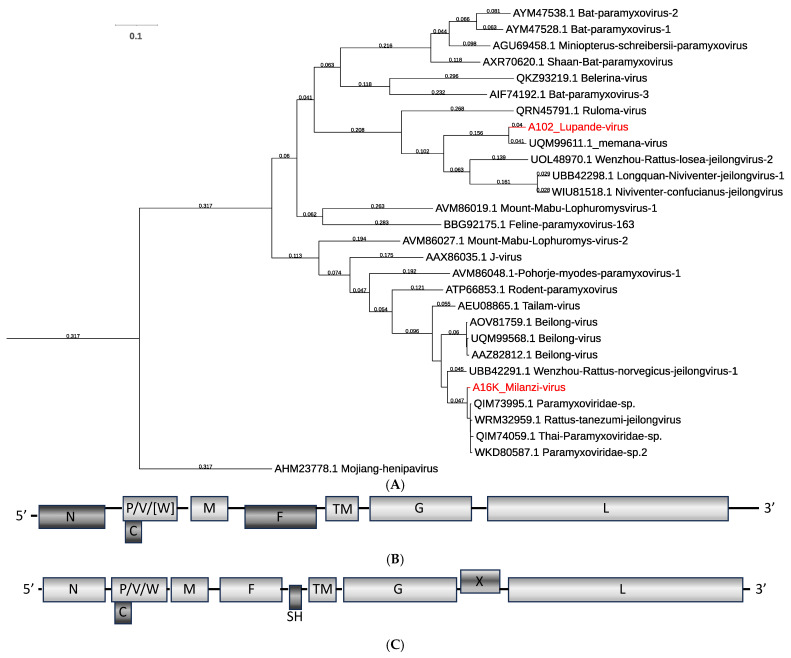
Jeilongviruses from Zambia. (**A**) Phylogenetic relationship of Milanzi and Lupande viruses to other viruses in the genus *Jeilongvirus* based on the polymerase amino acid sequence. Phylogeny was reconstructed using a Clustal W-aligned polymerase amino acid sequence (gap generation and extension penalties of 5 and 1, respectively) with the maximum likelihood method by applying a JTT model as implemented in IQtree; the best bootstrap tree from 500 pseudoreplicates is shown, and branch lengths ≥0.03 substitutions per site are indicated at the respective nodes. GenBank accession numbers are given next to the branches. The red font indicates viruses described in this study. (**B**) Schematic of Lupande virus genome organization. (**C**) Schematic of Milanzi virus genome organization. Gray shading indicates the three possible reading frames.

Short PCR-generated sequences in a previous study had already indicated the presence of several clades of paramyxovirus-related viruses in Zambian rodents [5]. Here we report the full genome coding sequence for one of these, as well as for a second, distantly related novel virus. Two contigs of approximately 20 kb were assembled that clustered in a phylogenetic analysis with members of the genus *Jeilongvirus* (subfamily *Orthoparamyxovirinae*, family *Paramyxoviridae*) (Figure 3A). The full coding sequence of the previously PCR-detected virus, Lupande virus, was related to sequence reported for memana virus from Guinea [10]. The sequence of the second virus, Milanzi virus, mapped to a distant clade related to Tailam and Beilong viruses. Both appear to represent novel species according to ICTV species demarcation criteria of a terminal branch length >0.03 [7]. Figure 3A also indicates that, together with Milanzi virus, several viruses reported from Southeast Asia belong to the same species, whereas Wenzhou rattus norvegicus jeilongvirus 1 would represent a separate species.

The genome organization of Lupande virus was compatible with other viruses in that clade, showing conserved transcriptional start/stop signals and sequence features to generate N, P (C, V, W), M, F, TM, G, and L proteins (Figure 3B, Supplementary Table S6). The putative editing site in P appears conserved and could generate a V protein that is similar to other jeilongviruses. The W ORF contains a stop after 17 amino acids, as in memana virus, and shows no homology to other proteins in the database so the function of such a protein remains questionable. The genome organization of Milanzi virus matched that of the other viruses in the clade, characterized by the additional SH and X ORFs (Figure 3C) [8,10].

In both Zambian sequences, the N ORF extends to the 5′-end of the generated sequences with several potential start codons. The amino acid sequence 5′ of M_40_ in Lupande virus does not match any entry in the GenBank database. In Milanzi virus, sequence 5′ of M_64_ does match other jeilongvirus sequences up to almost M_21_ (e.g., Beilong virus, GenBank OQ715595). It remains to be seen whether some newly identified jeilongviruses deviate from the better-studied viruses that include sequence data from purified viruses and/or rapid amplification of cDNA ends [RACE]. Given the conservation of transcriptional start sites in paramyxoviruses, it appears unlikely that sequence 5′ of M_40_ in Lupande virus or M_64_ in Milanzi virus is expressed (Supplementary Table S6).

Lupande virus was detected in 36 multimammate mice (31%, 36/118, 2–100% genome coverage). Milanzi virus was found in two black rats (2%, 2/118; 17% and 100% genome coverage) (Table 1).

### 3.4. Murine Aichivirus from Zambia

**Figure 4:**
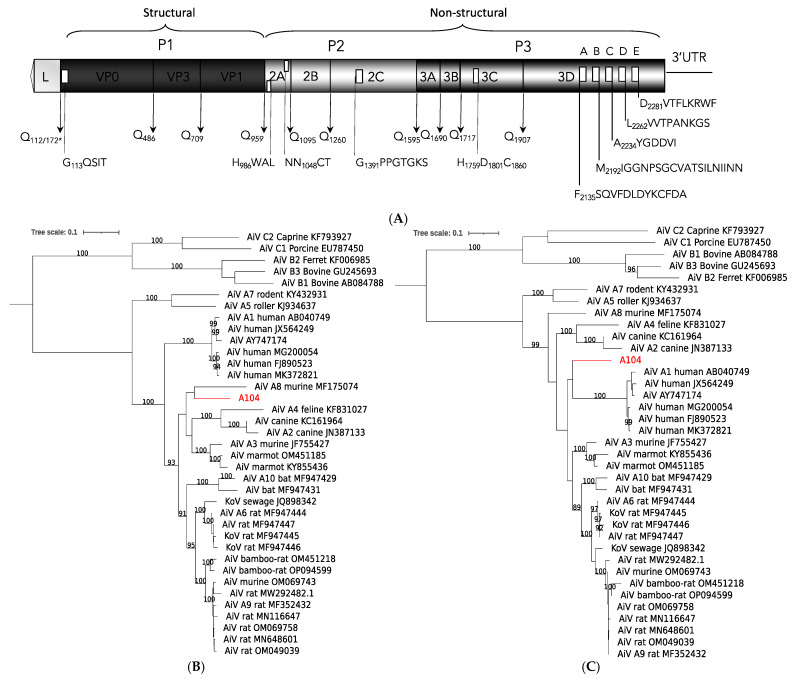
Murine kobuvirus A104 from Zambia. (**A**) Schematic of genome organization. Predicted polyprotein cleavage sites are indicated; * Q_112_ corresponds to Q_172_ in the complete leader sequences of aichivirus A3 or A8. The potential myristylation site in VP0, H-box/NC motif in 2A, helicase motif in 2C, protease triad in 3C, and polymerase domains A, B, C, D, and E in 3D are also indicated. Gray shading indicates different genome regions: leader, P1, P2, and P3. (**B**,**C**) Phylogenetic relationship to other viruses in the species *Aichivirus A* based on the polyprotein (**B**) and P1 amino acid sequence (**C**). Phylogeny was reconstructed with the maximum likelihood method by applying a LG+F+R4 substitution model as selected by Model Finder implemented in IQtree; bootstrap values (>85%) resulting from 1000 pseudoreplicates are indicated at the respective nodes; the scale bars indicate the number of amino acid substitutions per site, and GenBank accession numbers are given next to the branches. The red font indicates viruses described in this study.

A 7.3 kb sequence was assembled from a pool of kidney and lung of a multimammate mouse from Zambia that aligned with sequences of viruses in the species *Aichivirus A* (genus *Kobuvirus*, subfamily *Kodimesavirinae,* family *Picornaviridae*). The genomic sequence codes for a polyprotein with conserved protease cleavage motifs to generate mature proteins VP0, VP3, VP1, 2A, 2B, 2C, 3A, 3B, 3C, and 3D (Figure 4A). The sequence appears to have a gap of 10 amino acids in VP1, and we did not obtain a sequence for the first 70 amino acids of the leader when compared to aichivirus A3 (murine kobuvirus, USA; [38]) or A8 (murine kobuvirus, USA; [25]). Depending on the genome region, the sequence shows varying relationships possibly reflecting ancient recombination events (Figure 4B,C, Supplementary Figure S1, and Supplementary Table S7). Overall, it appears phylogenetically separated from the more consistently related cluster formed by A6, A9, and A10 viruses and closer to the variably branching murine A8 and A3 viruses. The Zambian virus did not match the currently recognized 10 viruses, or ‘types’, and would qualify as an additional virus or type in the species *Aichivirus A*.

A virtually identical sequence (6–71% genome coverage) was detected in 12 additional animals (11%, 13/118); all were multimammate mice (Table 1). To our knowledge, this is the first report of aichivirus A in African rodents, and it indicates the multimammate mouse as a native host of murine kobuvirus in Zambia.

## 4. Discussion

Rodents and shrews frequently come into contact with humans. It is critical to assess viral diversity and identify novel viruses circulating within such reservoir species. Rodent surveillance in Zambia and southern Africa is important, as exemplified by the still-unknown reservoir of Lujo virus. Although we did not find Lujo virus, our non-targeted approach (VirCapSeq-VERT) generated data that substantially extend our knowledge about circulating rodent viruses in Zambia and serve as a model for routine reservoir species surveillance for potential future zoonotic transmission events [48]. As strengthened by recent SARS-CoV, SARS-CoV-2, or influenza A virus H5N1 emergences, proper pre-pandemic surveillance must not only include exotic or novel viruses but also all animal species in direct and frequent contact with humans and/or production livestock.

Numerous examples of zoonotic transmission of hanta-, arena-, pox-, and other viruses exist. Prior publications have, for example, noted the presence of picorna-like calhevirus in the enteric virome of shrews [49]. Calheviruses have also been found in human stool [50], and while it is plausible that detection of viruses such as calhevirus in human stool is an incidental finding (due to consumption of food containing the virus), adaptation to new hosts upon continued viral exposure is possible so that monitoring in humans and contact animals appears prudent.

Our study showed aichivirus sequences in the organs of multimammate mice that represent a novel strain in the species *Aichivirus A*. Presence in organs with a substantial prevalence (11%) is more compatible with infection than incidental presence in non-sterile sites, e.g., in rectal swabs or feces via ingestion [29,51]. However, little is known regarding the pathology and tissue tropism of rodent aichiviruses. Previous studies have been performed mainly on fecal samples [25,30,31,33,34,35,36,37,38,52,53], and only four studies report tissue samples [25,32,35,36]. No overt pathology has been reported in any of the studies, as expected for reservoir hosts that are commonly not significantly affected by the infection. A potential for cross-species transmission of these viruses has been discussed based on phylogenetic analyses [30,35,54,55]. In ongoing VirCapSeq-VERT studies in Zambia of pediatric respiratory disease, pneumonia cases, and pediatric diarrhea cases, we found related aichivirus sequences in nasopharyngeal and stool samples (unpublished data). Human Aichi virus sequences have been found previously in Africa, in diseased children from Tunisia (genotype A), Ethiopia (no genotype reported), Burkina Faso (genotypes A, B, and C), and a single case from Nigeria (genotype B) [52,53,56,57]. Aichi virus has also been reported from environmental samples in Tunisia (genotypes A and B) and South Africa (no genotype reported) [53,58,59]. To our knowledge, this is the first report of an aichivirus A from African rodents, and it indicates the multimammate mouse as a prominent host species in Zambia. Considering the wide host species distribution—the species *Aichivirus A* currently includes viruses from humans, dogs, cats, mice, rats, bats, and birds—our data support a potential risk for zoonotic transmission.

The family *Paramyxoviridae* includes several important human pathogens, such as measles (genus *Morbillivirus*, subfamily *Orthoparamyxovirinae*) and mumps viruses (genus *Orthorubulavirus*, subfamily *Rubulavirinae*), as well as classical zoonotic viruses transmitted from fruit bat reservoirs, like Nipah or Hendra virus (both members of the genus *Henipavirus*, subfamily *Orthoparamyxovirinae*) [60]. Bats became an increasing focus of research after the SARS and MERS outbreaks and, more recently, the COVID-19 pandemic. However, rodents represent more zoonotic agents, have a larger diversity of species than bats, and are a prominent reservoir for viruses with pandemic potential [61,62]. Jeilongviruses form a young but rapidly growing genus in the subfamily *Orthoparamyxovirinae* that currently combines viruses found in rodents, cats, bats, shrews, hedgehogs, and tenrecs. Most of them share features such as an enlarged G ORF, accessory ORFs SH and/or TM, and where studied, a unique V protein-independent inhibition of signal transducer and activator of transcription (STAT) translocation in response to interferon activation [63]. Although the zoonotic potential of jeilongviruses remains unknown, some insights into biology and pathogenicity have been gained through studies on J-virus. J-virus can cause fatal disease in mice, and serosurveys have shown antibodies in mice, rats, pigs, a cow, and humans. However, experimental infection of pigs did not result in clinical disease, while neutralizing antibodies did develop in some infected animals [64,65]. Different isolates of J-virus exist; strain LW is not pathogenic in mice, and strain BH causes severe disease. Sequence analyses of recombinants indicated a role in J-virus pathogenesis for three single base mutations in which the two strains differ [66]. It has also been shown that the SH gene product is involved in pathogenicity; removal of SH in recombinant J-virus BH led to increased tumor necrosis factor alpha (TNF-α) production and apoptosis in vitro and to attenuation in mouse infections, similar to SH protein function in human mumps virus [67,68,69]. Milanzi virus includes an SH ORF, possibly linked to immune evasion and pathogenicity. Lupande virus matches previously reported short, PCR-derived L-gene sequences from Zambia and lacks the SH ORF. Combined with the previous study in Zambia [5], our data indicated that Lupande virus is widespread and highly prevalent. Lupande virus and the related memana virus [10] were found in the same mouse species, although in two distant regions of Africa (Zambia and Guinea). Of known jeilongviruses with the same genome organization and from countries neighboring Zambia, Ruloma virus from Tanzania [9] and Mount Mabu Lophuromys virus 1 and 2 from Mozambique [8] were found in the same rodent species, *Lophuromys machangui*, but only Ruloma virus maps in the same clade as Lupande virus (see Figure 3A). Milanzi virus branches in a monophyletic clade together with mainly short, PCR-derived sequences from Tunisia, Madagascar, and Reunion [70,71]. The clade also includes longer sequences from Asia, in part with gaps so that the overall genome organization remains unknown for some [72,73]. The current evolutionary grouping focused on the L gene sequence may not be optimal, as it does not consider pathogenicity factors (e.g., SH) and/or genes related to host specificity (G or F) [74,75,76]. We therefore favor a more differentiated, staged approach that takes additional genes and genome organization into account. Conserved L gene sequence may be appropriate for distant relationships at the family or subfamily level, but additional information from other genome regions may be added for genus demarcation criteria and full coding sequence considered for resolution at the species level.

MAdV-1 and -2 have been isolated from laboratory mice and only MAdV-3 from a striped field mouse (*Apodemus agrarius*) [15,16]. Pathology and tissue tropism for the three viruses are mainly known from experimental infections. MAdV-1 is primarily related to central nervous system (CNS) infection, but the virus also spreads to the kidney, spleen, and lung. However, outcomes vary with mouse strain, animal age, and route of infection [77,78]. MAdV-2-infected animals commonly appear healthy, and the virus is found in the intestinal tract and not in the lung, liver, or urine [15,16,79,80]. MAdV-3 was isolated from liver tissue, although the highest load was found in the lung of the striped field mouse [15]. It has been hypothesized for MAdV-1 that its residual pathogenicity might reflect an incomplete adaptation to the mouse host and a recent switch from an alternative host, while MAdV-2 is thought to be a genuine virus of mice that coevolved with its host. Little is known about murine adenoviruses in wild rodents. Small sequence fragments (~300 bp) obtained by nested PCR from feces have been reported from wild rodents in China (MAdV-2 [81] and MAdV-2, -1, -3 [31]) and by approaches in the USA (MAdV-2 [38]), and only two studies provide longer sequences (MAdV-1 [36] and MAdV-2 [25]). Similarly, MAdV-2-related, PCR-amplified sequences were reported from the dried blood spots of four rodents (*Cricetomys* (2), *Hybomys*, and *Praomys* spp.) in the Democratic Republic of the Congo, from the lung tissue of two multimammate mice in Kenya, and from the tissues of four rats in Côte d‘Ivoire [17,18,19]. In our study, we identified a novel fourth murine adenovirus in wild rodents, MAdV-4. Zoonotic transmission of MAdV-4 does not appear highly likely given the supposed species-specificity of adenoviruses and the sequence divergence from human mastadenoviruses [14]. However, there are reports that show or suggest transmission between human and non-human primates [82,83] and to and among animal species [12,19,84,85]. Recombination is an evolutionary mechanism in adenoviruses that may lead to sudden changes in host range, and findings of human adenoviruses in domestic animals support this notion [37,86,87]. However, recombination is not readily detected by common diagnostic assays that target one or a few genes of the virus, emphasizing the need for non-targeted, whole-genome detection approaches.

The genus *Chaphamaparvovirus* is composed of a rapidly growing group of related parvoviruses that have been identified in highly divergent species, including vertebrates and invertebrates. The type species *Rodent Chaphamaparvovirus1* includes two viruses, MuCPV from house mice trapped in New York City for which the disease state was not assessed [25] and MKPV, which was shown to cause inclusion body nephropathy (IBN) in laboratory mouse strains presumably worldwide [22]. The latter study also provided evidence that transcriptional activity and pathology are restricted to the kidney, although MKPV sequences can be found in other tissues like the liver or lung (possibly related to latent infection) [21,22,23,24]. Related viruses have been retrieved from kidney samples of vampire bats (*Desmodus rotundus*; Desmodus rotundus chapparvovirus, DrChPV) [22,88] and a capuchin monkey (*Cebus capucinus imitator*; capuchin kidney parvovirus, CKPV) [22], compatible with a more generalized nephro-tropism for these viruses. MKPV has been shown to express in the kidney a p10 protein at high levels from the most 5′ ORF of the genome. Conserved p10 ORFs are present in MuCPV, TdChPV2, and the virus a non-human primate, CKPV, but not in kidney-derived DrChPV from bats that map in an adjacent clade (see Figure 2). Given the relationship between these viruses and the diversity of hosts in which they have been found, it remains unclear to what extent these represent true virus reservoirs or incidental infection of susceptible hosts. As such, virus naming by species of encounter may be misleading. Both viruses from Zambia, Mwangazi virus and Nyamadzi virus, were present in kidney samples (Mwangazi virus was also in the lung), and both have a p10 ORF and map in a monophyletic clade together with MKPV, TdChPV2, CKPV, and UaPV (from the kidney of a black bear). For UaPV, only a 5′-truncated sequence is available that does not allow the identification of the (presumably present) p10 ORF [89]. Given the relationship between these viruses, phylogenetically and through a common p10 ORF, inter-species transmission appears conceivable and, in view of the likely nephro-tropic infection even in a non-human primate, of sufficient concern to include them in surveillance efforts.

Limitations of this study include the limited sample size and the lack of matching excreta. However, our study of Zambian rodents identified viruses from several diverse viral taxa that are, for various reasons, of potential zoonotic relevance. The applied VirCapSeq-VERT approach is a positive enrichment augmentation to next-generation sequencing that targets all viruses known to infect vertebrate hosts and thereby results in approximately 1000-fold increased sensitivity [39,40]. As shown, it is sample type- and pathogen-agnostic and capable of identifying partial or complete viral genomes, as well as novel sequences highly divergent from known sequences. It currently represents the most suitable approach to detect any virus in low copy number or present in poorly preserved samples, and thus presents a powerful tool for comprehensive pre-pandemic viral surveillance.

## Figures and Tables

**Table 1:** Paramyxo-, adeno-, parvo-, and picornaviruses identified in Zambian rodents.

Site *	Sample ID	Species ^#^	Tissue ^†^	Milanzi Virus	Lupande Virus	MAdV-4	Mwangazi Virus	Nyamadzi Virus	Aichivirus A
1	A104	MN	K/L		87 ^¶^		100		100
1	A121L	MN	L						53
1	A124K	MN	K/L		10				38
1	A131K	MN	K		100				68
1	A126K	MN	K/L				100		
1	A138	MN	K		3	99	100		
1	A13LV	MN	LV		2				9
1	A14	MN	K/LV		84		9		
1	A17L	MN	L				100		
1	A46L	MN	L		90				
1	A48K	MN	K		45	100			
1	A87L	MN	L						71
1	A93	MN	K		12	12			
1	A99L	MN	L				37		42
1	A102	MN	K/L		100				
1	A105	MN	K/L						9
1	A115	MN	K/L				15		
1	A127	MN	K/L		70	18			
1	A128	MN	K/L		93		100		
1	A24	MN	K/LV			11			40
1	A27	MN	L/LV				28		
1	A29	MN	K/LV		5				6
1	A30	MN	L/S				63		
1	A39	MN	L/S		4		100		
1	A58	MN	K/L		85		99		23
1	A59	MN	K/L		22				
1	A60	MN	K/LV		32				
1	A63	MN	L				43		
1	A64	MN	K		8		99		
1	A80	MN	L			12	98		
1	A83	MN	L		93		100		
1	A85	MN	K/L						43
1	A89	MN	K		3				
1	A95	MN	K		32	59	76		
1	A96	MN	K		27				
1	A97	MN	K		86				
1	A98	MN	K/L				37		
1	A119	MN	K/L		27				
1	A15	MN	L/LV		62				
1	A16K	RR	K	100				100	
1	A18	MN	K/L/LV				53		
1	A22	MN	L/S/K						67
1	A25	MN	K/L/LV			14			
1	A32	MN	K/L/LV/S		3				
1	A3	MN	K/LV/S		2			80	
1	A4L	MN	L		5				
1	A50	MN	K/L/LV				58		
1	A65	MN	K/L		92		100		
1	A67	MN	K/LV/S		2	5			
1	A69	MN	K/LV/S		4				
1	A6K	MN	K			3			
1	A72	MN	K/L		11				
1	A7	MN	L/LV/S				17		
2	K1	MN	K/L/LV		8				
2	K2	MN	K/L/LV		5				
2	K3	MN	K/L/LV		7				
2	K6	MN	K/LV/S		5				
3	NY2	RR	K/L/LV/S	17				100	
3	NY4	RR	K/L/LV/S					100	

* Trapping location: 1, 2, 3, or 4. ^#^ Species: MN = *Mastomys natalensis*; RR = *Rattus rattus*. ^†^ Tissue: K = kidney, L = lung, LV = liver, S = spleen; available nucleic acid extracts of an individual animal were pooled as indicated. ^¶^ Percent of genome coverage.

## Data Availability

Sequences are available at GenBank Accession numbers PQ450137 (aichivirus A11), PQ450138 (Lupande virus), PQ450139 (Milanzi virus), PQ490627 (Mwangazi virus), PQ490626 (Nyamadzi virus), and PQ490628 (murine adenovirus 4).

## Supplementary Materials

The following supporting information can be downloaded at: https://www.mdpi.com/article/10.3390/v16111754/s1, Figure S1: Phylogenetic relationship of murine kobuvirus A104 from Zambia to other viruses in the species Aichivirus A based on 2C3CD amino acid sequence; Table S1: Number of sequencing reads per sample; Table S2: Additional viruses identified in Zambian rodents; Table S3: Pairwise distance matrix for polymerase amino acid sequence of murine adenovirus from Zambia and other murine adenoviruses; Table S4: Conservation of splice sites in Mwangazi and Nyamadzi virus; Table S5: Pairwise distance matrix for NS1 amino acid sequence of Nyamadzi and Mwangazi virus, and other viruses in the genus *Chaphamaparvovirus*; Table S6: Deduced gene junctions and transcriptional editing site; Table S7: Pairwise distance matrix of murine kobuvirus from Zambia and other viruses in the species *Aichivirus A*.
