# Correction: Moonga et al. Application of a Sensitive Capture Sequencing Approach to Reservoir Surveillance Detects Novel Viruses in Zambian Wild Rodents. *Viruses* 2024, *16*, 1754

**DOI:** 10.3390/v17040561

**Published:** 2025-04-14

**Authors:** Lavel C. Moonga, Jones Chipinga, John P. Collins, Vishal Kapoor, Ngonda Saasa, King S. Nalubamba, Bernard M. Hang’ombe, Boniface Namangala, Tapiwa Lundu, Xiang-Jun Lu, Samuel Yingst, J. Kenneth Wickiser, Thomas Briese

**Affiliations:** 1 Department of Paraclinical Studies, School of Veterinary Medicine, University of Zambia, Lusaka 10101, Zambia; lavel.moonga@unza.zm (L.C.M.); mudenda68@yahoo.com (B.M.H.); b.namangala@unza.zm (B.N.); 2 Africa Centre of Excellence in Infectious Diseases of Humans and Animals (ACEIDHA), School of Veterinary Medicine, University of Zambia, Lusaka 10101, Zambia; 3 Vaughan Regional Medical Center, Selma, AL 36701, USA; chipingajoe2019@gmail.com; 4 Global Alliance for Preventing Pandemics at the Center for Infection and Immunity, Mailman School of Public Health, Columbia University, New York, NY 10032, USA; jc5966@cumc.columbia.edu (J.P.C.); vk2040@cumc.columbia.edu (V.K.); xl2134@columbia.edu (X.-J.L.); sy3229@cumc.columbia.edu (S.Y.); jkw2161@cumc.columbia.edu (J.K.W.); 5 Department of Zoology, Rabindranath Tagore University, Bhopal 464993, India; 6 Department of Disease Control, School of Veterinary Medicine, University of Zambia, Lusaka 10101, Zambia; nsaasa@gmail.com; 7 Department of Clinical Studies, School of Veterinary Medicine, University of Zambia, Lusaka 10101, Zambia; king.nalubamba@unza.zm; 8 Department of Biomedical Sciences, School of Veterinary Medicine, University of Zambia, Lusaka 10101, Zambia; tapiwalundu@gmail.com; 9 Department of Population and Family Health, Mailman School of Public Health, Columbia University, New York, NY 10032, USA; 10 Department of Epidemiology, Mailman School of Public Health, Columbia University, New York, NY 10032, USA

## Text Correction

The authors wish to make the following correction to this paper [1]:

There was an error in the original publication. In Section 3.2. “Rodent Chaphamaparvoviruses from Zambia”, the names ‘Mwangazi virus’ and ‘Nyamadzi virus’ have been switched in the following two sentences: “The region of D1 shows indel and sequence variation between the viruses, and two deduced D1 sites appear possible in the Mwangazi virus, the second one with better conservation (Supplementary Table S4). In the Nyamadzi virus, the analogous site does not conform with the canonical consensus, and another canonical site is located 5′ of the p10 termination codon so that a p10/p15 fusion protein would be generated that is not observed in the other viruses”.

A correction has been made to Section 3.2. in the first paragraph:

“We identified two 4 kb contigs with BLASTn homology of approximately 80% to murine chaphamaparvoviruses that branched in phylogenetic analyses separate from classified species (Figure 2A,B). Genomic features were analogous to those of mouse kidney parvovirus (MKPV; *Chaphamaparvovirus rodent1*), capuchin kidney parvovirus (CKPV, *Chaphamaparvovirus primate1*), and Tasmanian devil-associated chapparvovirus 2 (TdChPV2, *Chaphamaparvovirus dasyurid2*), which are species in the genus *Chaphamaparvovirus* (subfamily *Hamaparvovirinae*, family *Parvoviridae*). A common feature of these viruses is a 5′ p10 ORF that is also present in the viruses from Zambia. Based on NS1 and VP1 analyses, these viruses are also close to *Ursus americanus* parvovirus (UaPV; *Chaphamaparvovirus carnivoran3*) and *Ursus thibetanus ussuricus* chapparvovirus (UtPV; not classified) (Figure 2A,B), but detailed analysis is hindered by the 5′ truncated sequence for these viruses (GenBank Accession NC_077031 and OR779981). Both Zambian viruses, named Mwangazi virus and Nyamadzi virus, include the SF3 helicase family signature motifs Walker A, B, B’, and C in NS1 [43,44] and a domain of unknown function (DUF) 3648 described for NS1 of Brazilian bat chaphamaparvoviruses (Figure 2C,D) [45]. Like other chaphamaviruses, they lack a PLA2 domain in VP that is found in other parvovirus genera [46]. Two major splice donor sites (D1, D2) and three acceptor sites (A1–A3) have been experimentally mapped for MKPV [22]. In Mwangazi and Nyamadzi viruses, A1–A3 and D2 appear largely conserved. The region of D1 shows indel and sequence variation between the viruses, and two deduced D1 sites appear possible in the Nyamadzi virus, the second one with better conservation (Supplementary Table S4). In the Mwangazi virus, the analogous site does not conform with the canonical consensus, and another canonical site is located 5′ of the p10 termination codon so that a p10/p15 fusion protein would be generated that is not observed in the other viruses (D1a, Supplementary Table S4). The region between D1 and A1 differs in additional aspects between the viruses. NS2-P and p15 in Mwangazi and Nyamadzi viruses constitute one continuous ORF, and expression of p15 functionality may not require efficient D1/A1 splicing, whereas splicing may be essential in the other viruses where NS2-P and p15 are in different frames (MKPV, CKPV) or separated by a stop codon (MuCPV, TdChPV2). Based on the NS1 amino acid identity of 74% between each other and less than 85% with the existing species, both viruses qualify as novel species in the genus *Chaphamaparvovirus* (Supplementary Table S5) [47].”

## Error in Figure/Table

In the original publication [1], there was a mistake in Figure 2 as published. In panels C and D, the names ’Mwangazi virus’ and ’Nyamadzi virus’ have been switched and associated with the opposite schematic. The corrected version of Figure 2 appears below.

**Figure 2:**
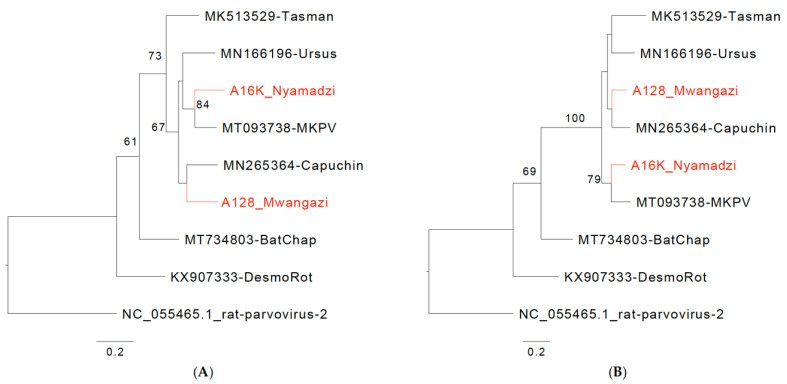

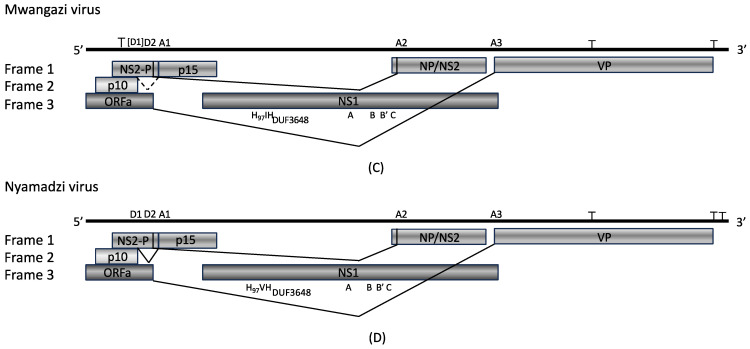
Chaphamaparvoviruses from Zambia. Phylogenetic relationship of Mwangazi and Nyamadzi viruses to other viruses in the genus *Chaphamaparvovirus* based on NS1 (**A**) and VP (**B**) amino acid sequences. Phylogeny was reconstructed with the maximum likelihood method by applying a JTT+G4 substitution model for NS1 and a Q.yeast+F+G4 substitution model for VP1, selected by Model Finder implemented in IQtree; bootstrap values (>60%) resulting from 1000 pseudoreplicates are indicated at the respective nodes; the scale bars indicate the number of amino acid substitutions per site, and GenBank accession numbers are given next to the branches. The red font indicates viruses described in this study. (**C**) Schematic of Mwangazi virus genome organization. (**D**) Schematic of Nyamadzi virus genome organization. Gray shading indicates the three possible reading frames. Predicted major splice sites (donor sites D1, D2, and acceptor sites A1–A3), polyadenylation signals (T), SF3 helicase (H97), Walker A, B, B’, C, and domain of unknown function (DUF) 3648 motifs are indicated.

In the original publication [1], there was a mistake in Supplementary Table S4 as published. The names ’Mwangazi virus’ and ’Nyamadzi virus’ have been switched. The corrected version of Supplementary Table S4 appears below.

**Table S4:** Conservation of splice sites in Mwangazi and Nyamadzi virus.

Canonical donor consensus	mAG GTr	mAG GTr	
MKPV	D1 GAAGGAG GTGAGTCAG	D2 GCCGAAG GTAATTAAA	
CKPV	D1 GAAAGAG GTGAGTCGC	D2 CCTGAAG GTACTTATC	
Nyamadzi virus	D1 GGTGGAG GTGAGGGAG	D2 GCGGAAG GTACTTATT	
Mwangazi virus	D1 GCAGAAG GAGAATCGG	D2 CCAGAAG GTACTTATT	
	D1a CCACAAG GTGCGAAAA		
Canonical acceptor consensus	cAG Gk	cAG Gk	cAG Gk
MKPV	A1 CTTCTTACAG ATGTCTAT	A2 TTATTTGCAG AGCTAGTG	A3 TTATTTACAG AAACACTA
CKPV	A1 ATGCATGCAG ATGTCTAT	A2 TCTTTTGCAG AACTAGTG	A3 TTATTTACAG CAACAATA
Nyamadzi virus	A1 TAATTTACAG ATGTCTCT	A2 TTATTTGCAG AGCTAGTG	A3 TCATTTACAG AAACAATA
Mwangazi virus	A1 TCATCTACAG ATGTCTAT	A2 TTGTTTGCAG AATTAGTG	A3 TTATTTGCAG AACAAATA

The authors state that the scientific conclusions are unaffected. The corrections were approved by the Academic Editor. The original publication has also been updated. The authors apologize for any inconvenience caused to the readers by these changes.
