# Supplementary material for: Application of a Sensitive Capture Sequencing Approach to Reservoir Surveillance Detects Novel Viruses in Zambian Wild Rodents
Source: Viruses. 2024 Nov 9;16(11):1754. doi: 10.3390/v16111754 (PMC11598836; doi:10.3390/v16111754)
Supplement: Supplementary file 1 [file viruses-16-01754-s001.zip › Supplementary Figure S1.pdf]

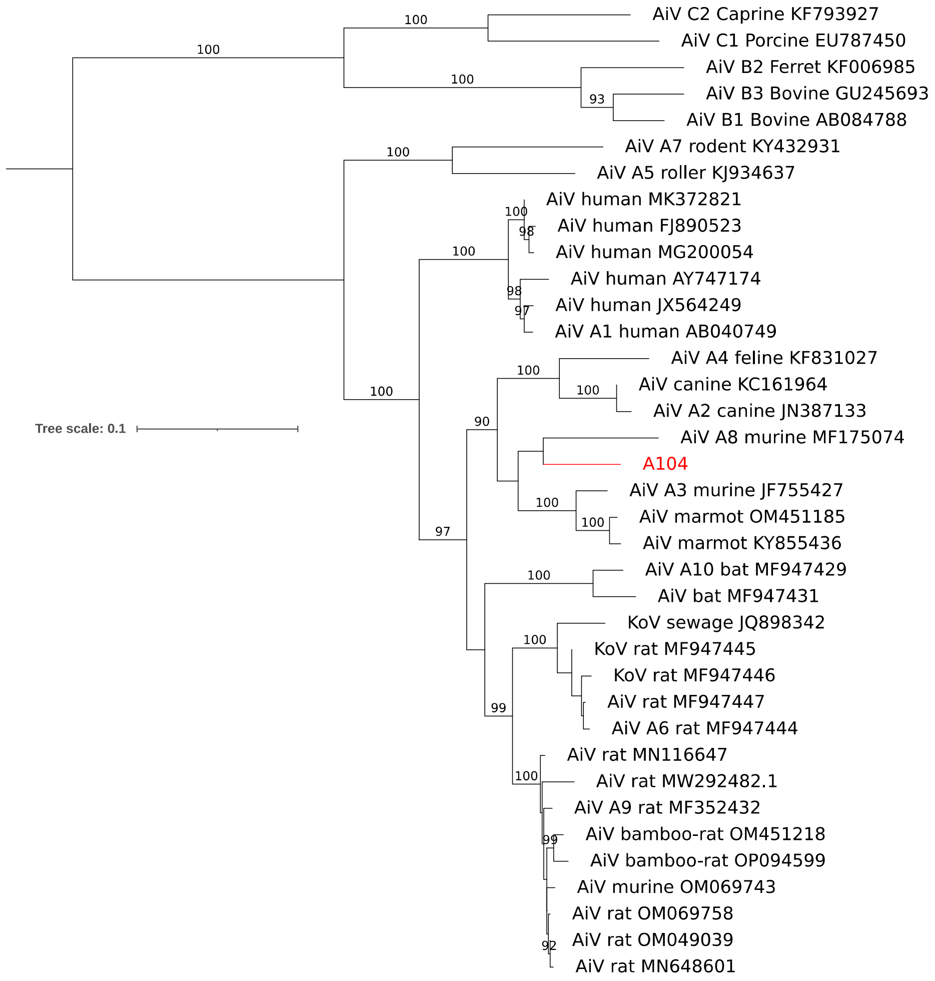

Supplementary Figure S1. Phylogenetic relationship of murine kobuvirus A104 from Zambia to other viruses in the species *Aichivirus A* based on 2C3CD amino acid sequence.
