# Supplementary material for: Application of a Sensitive Capture Sequencing Approach to Reservoir Surveillance Detects Novel Viruses in Zambian Wild Rodents
Source: Viruses. 2024 Nov 9;16(11):1754. doi: 10.3390/v16111754 (PMC11598836; doi:10.3390/v16111754)
Supplement: Supplementary file 1 [file viruses-16-01754-s001.zip › Supplementary Table S4.pdf]

**Supplementary Table S4**. Conservation of splice sites in Mwangazi and Nyamadzi virus.

| Canonical donor consensus | | mAG GTr | | mAG GTr |
|---|---|---|---|---|
| MKPV | D1 | GAAGGAG GTGAGTCAG | D2 | GCCGAAG GTAATTAAA |
| CKPV | D1 | GAAAGAG GTGAGTCGC | D2 | CCTGAAG GTACTTATC |
| Nyamadzi virus | D1 | GGTGGAG GTGAGGGAG | D2 | GCGGAAG GTACTTATT |
| Mwangazi virus | D1 | GCAGAAG GAGAATCGG | D2 | CCAGAAG GTACTTATT |
| | D1a | CCACAAG GTGCGAAAA | | |

| Canonical acceptor consensus | | cAG Gk | | cAG Gk | | cAG Gk |
|---|---|---|---|---|---|---|
| MKPV | A1 | CTTCTTACAG ATGTCTAT | A2 | TTATTTGCAG AGCTAGTG | A3 | TTATTTACAG AAACACTA |
| CKPV | A1 | ATGCATGCAG ATGTCTAT | A2 | TCTTTTGCAG AACTAGTG | A3 | TTATTTACAG CAACAATA |
| Nyamadzi virus | A1 | TAATTTACAG ATGTCTCT | A2 | TTATTTGCAG AGCTAGTG | A3 | TCATTTACAG AAACAATA |
| Mwangazi virus | A1 | TCATCTACAG ATGTCTAT | A2 | TTGTTTGCAG AATTAGTG | A3 | TTATTTGCAG AACAAATA |
