# Supplementary material for: Application of a Sensitive Capture Sequencing Approach to Reservoir Surveillance Detects Novel Viruses in Zambian Wild Rodents
Source: Viruses. 2024 Nov 9;16(11):1754. doi: 10.3390/v16111754 (PMC11598836; doi:10.3390/v16111754)
Supplement: Supplementary file 1 [file viruses-16-01754-s001.zip › Supplementary Table S6.pdf]

**Supplementary Table S6**. Deduced gene junctions and transcriptional editing site.

| Milanzi | | | | Lupande | | | |
|---|---|---|---|---|---|---|---|
| | Stop | IGR* | Start | | Stop | IGR* | Start |
| N | | [CAT] | AGGAGCAAAG | N | | [CTT] | AGGAGAAAAG |
| N/P | ATTAAGAAAAA | CTT | AGGAACAAGG | N/P | ATTAAGAAAAA | CTT | AGGATCAATG |
| P/M | ATTAAGAAAAA | CTT | AGGGCCAAGG | P/M | ATTAGAAAAAA | CTT | AGGGTGAAAG |
| M/F | TCTAGAAAAAA | CTT | AGGTAGAAGG | M/F | ATTAAGAAAAA | CTT | AGGGATAAAG |
| F/SH | ATTAGAAAAAA | CTT | AGGGTGACAG | F/TM | ACAATAAAAAA | CTT | AGGAGAAATG |
| SH/TM | ACAATAAAAAA | CTT | AGGTTTAATG | TM/G | ATAATAAAAAA | CTT | AGGAGAAATG |
| TM/G | ATTACAAAAAA | CTT | AGGGTCAAGG | G/L | TTTATAAAAAA | CTT | AGGAACAAAG |
| G/(X) | -/- | -/- | -/- | L | ATTAAGAAAAA | [CTT] | |
| G(X)/L | GTTAAGAAAAA | CTT | AGGACGAATG | | | | |
| L | ATTAAGAAAAA | [CTT] | | | | | |

*IGR - intergenic region.

## Consensus sequences

| | | | | | | | |
|---|---|---|---|---|---|---|---|
| Milanzi | DWWAnRAAAAA | CTT | AGGDnBAMDG | Lupande | WYWADRAAAAA | CTT | AGGRDnAAWG |
| Wenzhou | DYWADRAAAAA | CTT | AGGRnVAAWG | Memana | DTWADRAAAAA | CTT | AGGRDHAAHG |
| Tailam | WYWADRRAAAA | CTT | AGGRnBAAWG | Mossman | MYWAHRAAAAA | CKT | AGGRBnMARG |

## P gene editing site

| | | | |
|---|---|---|---|
| Milanzi | TGTTAAAAAAGGCA | Lupande | GTTGTTAAAAGGGGCA |
| Wenzhou | TGTTAAAAAAGGCA | Memana | GTTGTTAAAAGGGGCA |
| Tailam | AGTTAAAAAAGGCA | Mossman | CAGACTAAAAGGGGCA |
